# Increasing Fracture Toughness and Transmittance of Transparent Ceramics using Functional Low-Thermal Expansion Coatings

**DOI:** 10.1038/s41598-018-33919-5

**Published:** 2018-10-23

**Authors:** Marc Rubat du Merac, Martin Bram, Jürgen Malzbender, Mirko Ziegner, Marcin Rasinski, Olivier Guillon

**Affiliations:** 1 Forschungszentrum Jülich GmbH, Institute of Energy and Climate Research (IEK), 52425 Jülich, Germany; 2 Geomaterialwissenschaft, Institut für Angewandte Geowissenschaften, Technische Universität Darmstadt, 64287 Darmstadt, Germany; 3 Jülich Aachen Research Alliance: JARA-Energy, 52062, Aachen, 52425 Jülich, Germany

## Abstract

Transparent polycrystalline ceramics have the potential to enable applications no other materials can, but to do so their strength and toughness must be improved. However, surface strengthening treatments like those used for glasses have so far remained elusive. Here for the first time, we report on engineering unprecedented surface compression, of the magnitude achieved for ion-exchange strengthened glasses (~750 MPa) in transparent ceramics. This was achieved by applying functional, low thermal-expansion yttria coatings onto yttria-stabilized zirconia substrates and thermally treating. In some instances, the treatment more than doubled the fracture toughness while simultaneously increasing light transmittance.

## Introduction

Polycrystalline transparent ceramics have unique properties that can enable applications few other materials can; for example windows and lenses for ultraviolet lithography and infrared imaging, laser host materials, scratch and heat resistant windows, and lightweight transparent armor^1–5^. However, they must be sintered at high temperatures to near-theoretical density while maintaining a homogeneous, optically-isotropic crystal structure, which limits solute addition possibilities and complicates processing^1,2^. Moreover, transparent ceramics can develop inter-granular porosity and secondary phases, can undergo polymorphic transitions, and are extremely sensitive to impurities, features which can cause light scatter and absorption^1,2,6,7^. These constraints render transparent ceramics more expensive to manufacture than glasses and glass-ceramics, and although their strength can be greater^1,8,9^, achieved values remain far below theoretical limits due to remaining defects^10^. One new approach developed for tellurite-based transparent ceramics combined established glass-forming with subsequent complete crystallization of net-shaped parts and all processing performed at intermediate temperatures below 1,000 °C^5^. A similar approach was successfully applied for the synthesis of transparent yttrium aluminium garnet-based nanoceramics starting from Y_2_O_3_-Al_2_O_3_ bulk glasses^11^. However, such methods are not viable for all systems and they do not address improving the fracture toughness. Recently, Nishiyama *et al*. reported the synthesis of transparent polycrystalline cubic silicon nitride, which is promising regarding its mechanical properties and application under severe conditions, but processing requires ultra high pressure equipment (pressure above 13 GPa) to stabilize the cubic phase^12^. Another highly promising approach to further increase mechanical and optical properties of transparent ceramics is the implementation of surface compression. While glasses are strengthened by inducing surface compression by tempering or ion-exchange, analogous surface treatments for transparent ceramics have generally achieved only moderate surface compression, and even this only for opaque ceramic laminates^13–20^. However, surface compression can in principle also be induced by exploiting thermal-expansion mismatch between different materials or material stoichiometries^13^. Yttria-stabilized zirconia (YSZ) is one promising transparent ceramic amenable to such a treatment^21^. It has exceptional physical properties and potential for use as miniaturized lenses and protective windows and domes^21–23^. In the present work, we prove the feasibility of this novel concept by implementing high surface compression in transparent YSZ by applying functional, low-thermal expansion yttria thin-coatings followed by a thermal treatment.

## General Concept

Ceramics typically fail at existing stress-concentrating flaws near surfaces, where tensile loading stresses are usually highest, for example during bending^13,24^. Surface compression acts against crack-initiating and opening during loading, thus strengthening and toughening by requiring higher stress to activate flaws. Surface compression can be generated by cooling two bonded materials, or material stoichiometries, with different thermal expansion from elevated temperature. The material with greater thermal expansion shrinks more, imparting compressive stress in the other. If the material with lower thermal expansion is a thin coating, significant compressive stress (*σ*_C_) can be achieved (Eq. 1);

1 $${\sigma }_{{\rm{C}}}=-\frac{\varepsilon {E^{\prime} }_{{\rm{C}}}}{1+({t}_{{\rm{C}}}{E^{\prime} }_{{\rm{C}}}/{t}_{{\rm{S}}}{E^{\prime} }_{{\rm{S}}})}$$

which is balanced by moderate tensile stress (*σ*_S_) in the substrate (Eq. 2);

2 $${\sigma }_{{\rm{S}}}=-\,{\sigma }_{{\rm{C}}}\frac{{t}_{{\rm{C}}}}{{t}_{{\rm{S}}}}$$

where *t* is thickness, *E* elastic modulus, the subscripts “c” and “s” refer to coating and substrate, and *ε* is strain **(**Eq. 3**)**;

3 $$\varepsilon ={\int }_{{{\rm{T}}}_{{\rm{C}}}}^{{{\rm{T}}}_{{\rm{H}}}}({\alpha }_{{\rm{S}}}-{\alpha }_{{\rm{C}}}){\rm{dT}}\approx ({\alpha }_{{\rm{S}}}-{\alpha }_{{\rm{C}}})({{\rm{T}}}_{{\rm{H}}}-{{\rm{T}}}_{{\rm{C}}})$$

where *α*_S_ and *α*_C_ are coefficients of thermal expansion (CTE), and *T*_H_ and *T*_C_ are the heating and cooling temperatures^18^.

### Example

**Figure 1:**
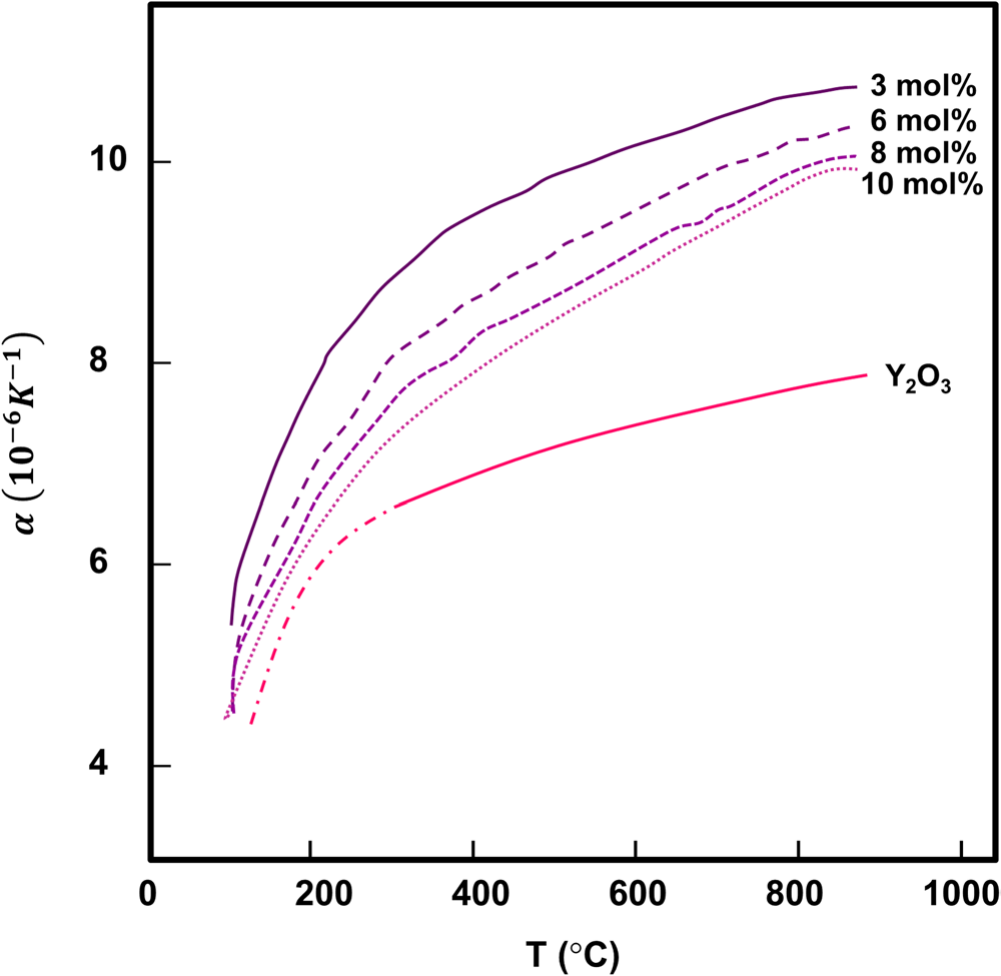
Thermal expansion of YSZ as a function of Y_2_O_3_ content (adapted with permission, 2005, Elsevier)^30,43^.

Pure zirconia exists as monoclinic (<950 °C), tetragonal (1200–2370 °C), and cubic (>2370 °C) polymorphs^25,26^, the former being undesirable due to birefringence^27^. The optically-isotropic cubic phase is retained to room temperature by replacing Zr^4+^ with aliovalent cations (Mg^2+^, Ca^2+^, Ce^3/4+^, and Y^3+^) that stabilize the fluorite structure by generating oxygen vacancies^24^. Addition of 3–7 mol% yttria (Y_2_O_3_) stabilizes the cubic and tetragonal phases, whereas >8 mol% nearly or completely stabilizes the cubic phase^26,28^. Solid-solution exists up to 40 mol% Y_2_O_3_, at which the only intermediate compound Zr_3_Y_4_O_12_ forms^29^. Cubic yttria-stabilized zirconia (YSZ) exhibits high transmission from the near-ultraviolet to the mid-infrared, a high refractive index, optical isotropy, and low emissivity combined with chemical resistance, low thermal conductivity, and high hardness, strength, and toughness^1,26^. Moreover, its CTE decreases with increasing yttria content (Fig. 1)^30^, while retaining the cubic structure and transparency over a wide range, enabling thermal-expansion mismatch toughening by varying stoichiometry.

Although surface compression could be induced by varying YSZ stoichiometry at surfaces, using the cubic Y_2_O_3_ end-member would yield the highest stress. Substituting values for a 750 nm thick Y_2_O_3_ coating bonded to an 8 YSZ substrate into Eqs 1–3, surface compression of ~850 MPa should be achievable. Significantly, this would be of the same magnitude as stresses in ion-exchanged strengthened glass^31–33^. However, making transparent YSZ substrates with Y_2_O_3_ surfaces requires optically-planar interfaces since the refractive indices are different. Moreover, post-polishing would add cost, complicate obtaining the desired thickness, and introduce surface flaws. These constraints can be overcome by first making polished transparent substrates and subsequently applying coatings followed by thermal treatment.

## Methods

Transparent YSZ substrates were made by cold-pressing 8 and 10 mol% YSZ powders (TZ-8Y, 10Y, Tosoh Corp., Tokyo, Japan and DSZ-64 [10Y], Daiichi Kigenso Kogyo Co., Osaka, Japan) at 50 MPa in 20 mm diameter stainless steel dies, cold-isostatic pressing at 300 MPa (Type SI4-0 3-3, Dieffenbacher, Eppingen, Germany), pressureless sintering at 1260 °C for 20 h in air (HT 160/16, Nabertherm, Lilienthal, Germany), and hot isostatic pressing at 1450 °C for 1 hour in argon (Engineered Pressure Systems International [EPSI], Temse, Belgium)^22^. The ~2.5 mm thick substrates were ground and polished to an optical finish with SiC grinding papers, Al_2_O_3_ suspensions to 1 μm, and colloidal silica. Yttria coatings 0.5–1.5 μm thick were deposited by electron-beam physical vapor deposition (EB-PVD) (CS400ES, Von Ardenne, Dresden, Germany) using cold-pressed yttria targets (Grade C > 99.95%, H.C. Starck, Goslar, Germany), a substrate temperature of 750 °C, vacuum with 5 cm^3^/min O_2_, and coating thickness determined using an integrated quartz-crystal microbalance and post-deposition Calo-test. Coated and uncoated substrates were annealed in argon atmosphere at 1450 °C for 1–12 hours (Nabertherm) and quenched in a furnace having relatively rapid natural cooling (approximately 10 °C/min down to 800 °C).

Coated and uncoated substrates were examined by optical microscopy (Axiovert.A1, Zeiss, Oberkochen, Germany) and scanning electron microscopy (SEM) (TM3030, Hitachi, Tokyo, Japan; Ultra-55, Zeiss) in combination with energy-dispersive X-ray spectroscopy (EDS). Grain sizes were determined from optical and SEM images using the circle-intercept method. Focused ion-beam (FIB) cross-sections (NVision 40 Cross-Beam Workstation, Zeiss) were examined by transmission electron microscopy (TEM) (Tecnai G2 F20, FEI, Hillsboro, OR, USA).

Phase and stress analysis were performed using an X-ray diffractometer equipped with a Eulerian cradle and Cu-Kα source (Empyrean, PANalytical, Almelo, The Netherlands). For qualitative phase analysis, a BBHD-mirror, 0.02-rad Soller slit, 2° anti-scatter slits, and a PIXcel3D detector were used. Peak positions and lattice parameters were determined by Rietveld refinement using TOPAS software (Bruker AXS, Billerica, MA, USA) and matched using HighScore Plus (PANanalytical) and PDF-2 database (ICDD, Newton Square, PA, USA). Stress analysis and pole figures were made using a parallel-beam X-ray lens with crossed-slits assembly, Ni Kβ filter, 0.18° parallel-plate collimator, and a proportional detector. Residual stress was determined using the sin^2^ψ method and Stress Plus software (PANanalytical) and magnitudes determined from the slope of least-squares fits of *d* versus sin^2^ψ plots using Eq. 4, where *E* is the modulus, *ν* the Poisson’s ratio, and *m* the slope.

4 $${\sigma }_{{\rm{C}}}=(E/1+{\nu }){m}$$

Several diffraction set-ups and sets of diffracting planes for the substrate (311, 620) and coating (222, 400, 622) were used to optimize and validate measurements. Results were based on Y_2_O_3_ (622) and YSZ (620) reflections and elastic moduli and Poisson’s ratios of 171.5 GPa and 0.298 and 215 GPa and 0.290 for Y_2_O_3_ and YSZ, respectively^34,35^.

Hardness and toughness were determined by indentation using a Vickers hardness tester (Duramin-A300, Struers, Ballerup, Denmark) and 200, 300, and 500 g loads. Apparent fracture toughness values were calculated using the Anstis relation (Eq. 5)^36^;

5 $${K}_{{\rm{IC}}}=0.016{(E/H)}^{1/2}(P/{{c}}^{3/2})$$

where 0.016 is a correction for half-penny shaped radial cracks typically observed in YSZ, *E* is the modulus, *H* is Vickers hardness, *P* is the hardness testing load, and *c* is the indentation crack length measured by optical microscopy and SEM.

In-line and total transmittance and diffuse and total reflectance were measured using a spectrophotometer equipped with an integrating sphere (Lambda 950, Perkin-Elmer, Waltham, MA, USA). Forward scatter, specular reflectance, and absorptance were calculated by difference from Eq. 6;

6 $${{I}}_{{I}}=({{I}}_{{DR}}+{{I}}_{{SR}})+{{I}}_{{A}}+({{I}}_{{DT}}+{{I}}_{{ILT}})$$

where the subscripts *I* refer to incident, *DR* to diffuse reflected, *SR* to specular reflected, *A* to absorbed, *DT* to diffuse transmitted, and *ILT* to in-line transmitted intensities.

## Results

**Figure 2:**
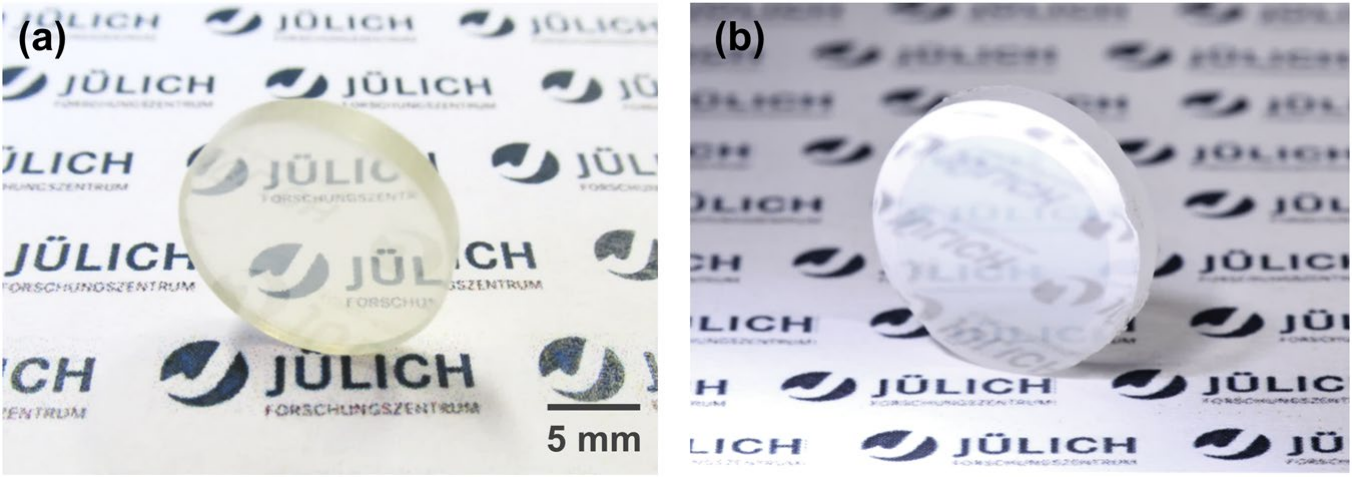
Initial transparent 10 YSZ compact (**a**), and after coating with 0.50 μm of Y_2_O_3_ (**b**) (incident light oriented to reflect and highlight coating, see also Fig. 8).

**Figure 3:**
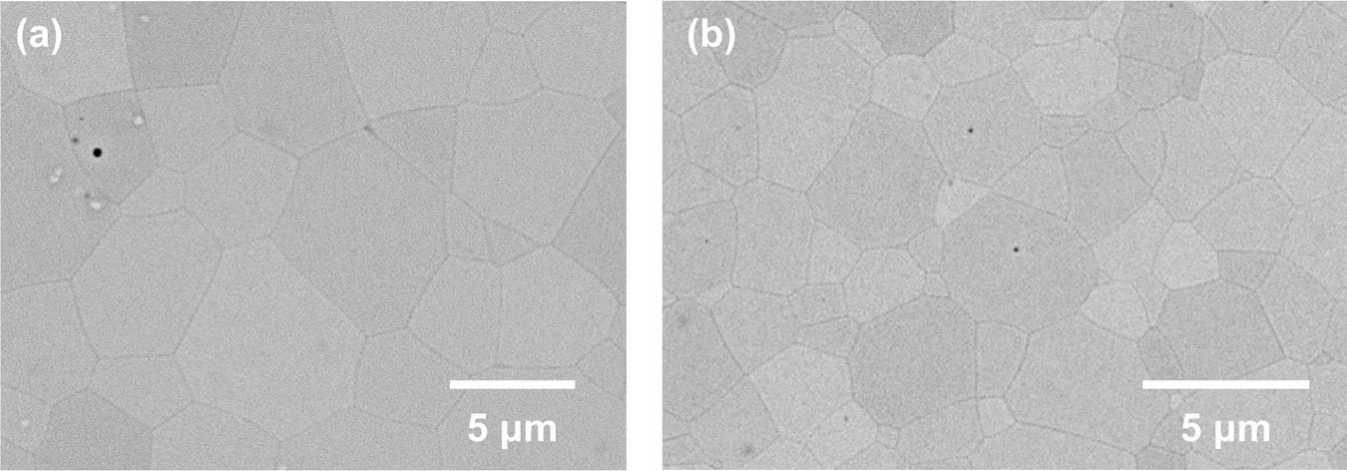
Microstructure of uncoated 8 YSZ (**a**) and 10 YSZ (**b**) substrates (SEM).

Pressureless sintered and hot-isostatically pressed 10 YSZ substrates (Fig. 2a) were fully-dense and transparent and remained so after applying yttria coatings (Fig. 2b). Transparency was lower for thicker coatings and for 8 YSZ substrates, the latter consistent with comparatively lower cubic phase stabilization. Average grain sizes were 5.4 μm and 1.6 μm for 8 YSZ and 10 YSZ substrates, respectively (Fig. 3), with differences attributed to starting powder differences^37^. Occasional small (<500 nm) residual occluded pores (Fig. 3) likely formed due to rapid grain growth during hot-isostatic pressing.

**Figure 4:**
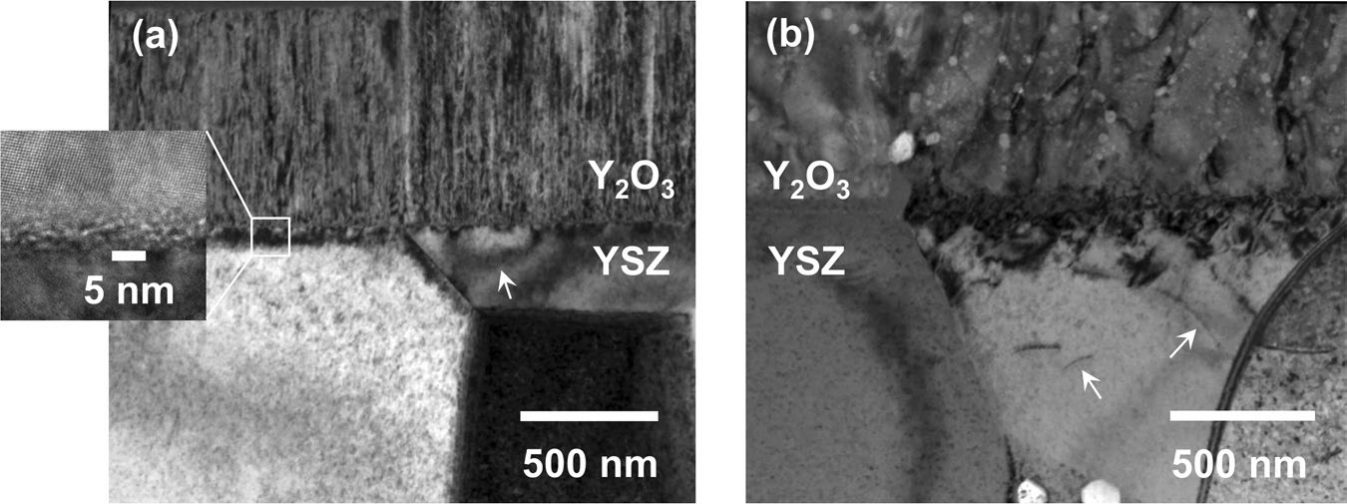
(**a**) As-deposited 0.75 μm thick Y_2_O_3_ coating on an 8 YSZ substrate and **(b)** after annealing at 1450 °C in argon for 1 h followed by quenching (TEM; arrows indicate strain-contours and dislocations and bright spots in **(b)** correspond to ion beam damage).

As-deposited yttria coatings were fully-dense and exhibited a columnar morphology (Fig. 4a). Coating-substrate interfaces were planar with a <5 nm thick disordered zone. Strain contours observed in the underlying YSZ substrates suggested residual stress. Grain growth occurred in coatings after thermal treatment at 1450 °C for 1 h in argon but the interface remained planar and dislocations in the substrate suggested the development of appreciable residual stress (Fig. 4b). Due to their sizes, the interface and underlying dislocations were not expected to affect optical properties in the visible range^38^.

**Figure 5:**
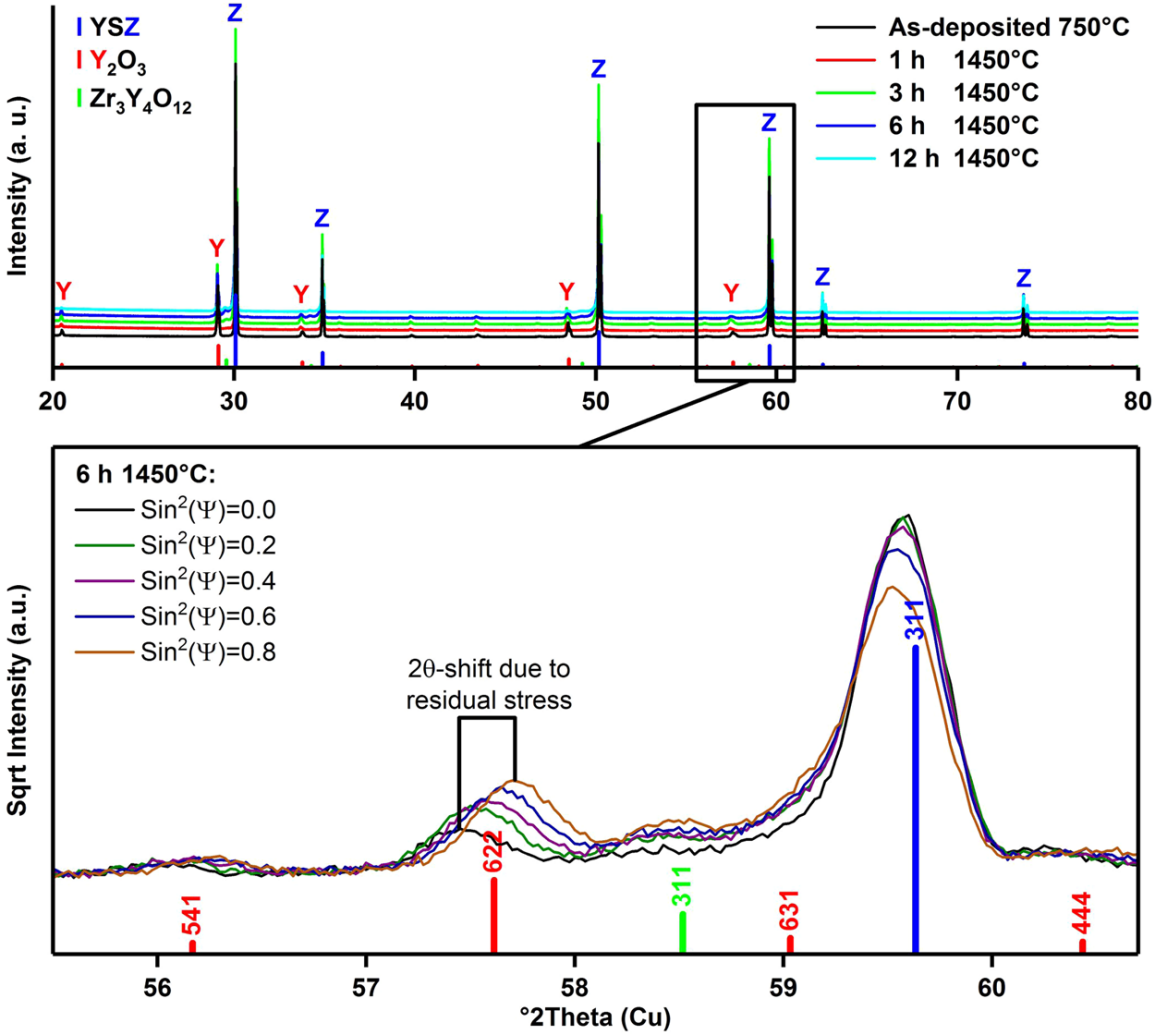
(top) XRD results for 0.75 μm Y_2_O_3_ EB-PVD coating on 8 YSZ substrate for different annealing conditions, (bottom) example of stress measurement at Y_2_O_3_ (622) reflection under various tilt angles (sample annealed at 1450 °C for 6 h). Reference powder patterns: ICSD 90894, Zr_0.79_Y_0.21_O_1.89_, Fm-3m, *a* = 5.1378 Å (blue); ICSD 26190, Y_2_O_3_, Ia-3, *a* = 10.6040 Å (red); ICSD 181238, Zr_0.4_Y_0.6_O_1.7_, Fm-3m, *a* = 5.227 Å (green); note: out of simplicity and due to uncertainty in the composition, this compound is referenced as Zr_3_Y_4_O_12_ in the figure and in the text.

**Figure 6:**
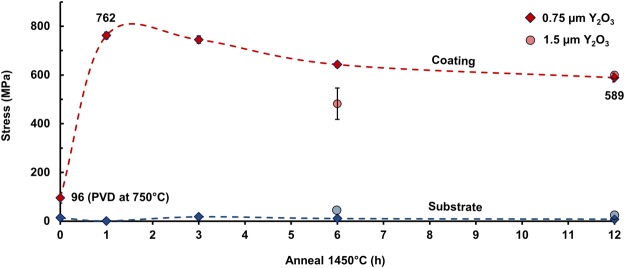
8 YSZ substrate and coating residual stresses as a function of annealing time at 1450 °C.

Only reflections corresponding to YSZ and yttria were indicated by X-ray diffraction (XRD) for coated 8 YSZ substrates (Fig. 5). However, for longer annealing times of 6 or 12 h, weak reflections corresponding to the intermediate cubic compound Zr_3_Y_4_O_12_ were noted (Fig. 5 inset). Pole figures (Fig. S1) indicated no texture for the YSZ substrates, but yttria coatings were preferentially oriented along {111} directions, consistent with other reports^39^. Plots of *d* versus sin^2^ψ were linear, indicating regular behavior with zero *ε*_13_ and *ε*_23_ (Fig. S2)^40^. Coatings exhibited moderate (~100 MPa) residual compressive stresses after deposition, balanced by moderate substrate tensile stresses (Fig. 6), as predicted by Eqs 1 and 2. Occasional indications of slight compressive stresses in substrates were attributed to yttria enrichment near the coating-substrate interfaces and XRD probing the near-interface region; stresses deeper within substrates were expected to be moderately tensile. Coating compressive stresses were significant (>500 MPa) only after thermal treatment at 1450 °C, consistent with expectations based on Eq. 3, and were highest for shorter annealing times, on the order of ~750 MPa for a 1 h anneal. Stresses were somewhat smaller for thicker coatings, as expected from Eqs 1 and 2. Similar behavior was expected for coated 10 YSZ substrates based on toughness results.

**Figure 7:**
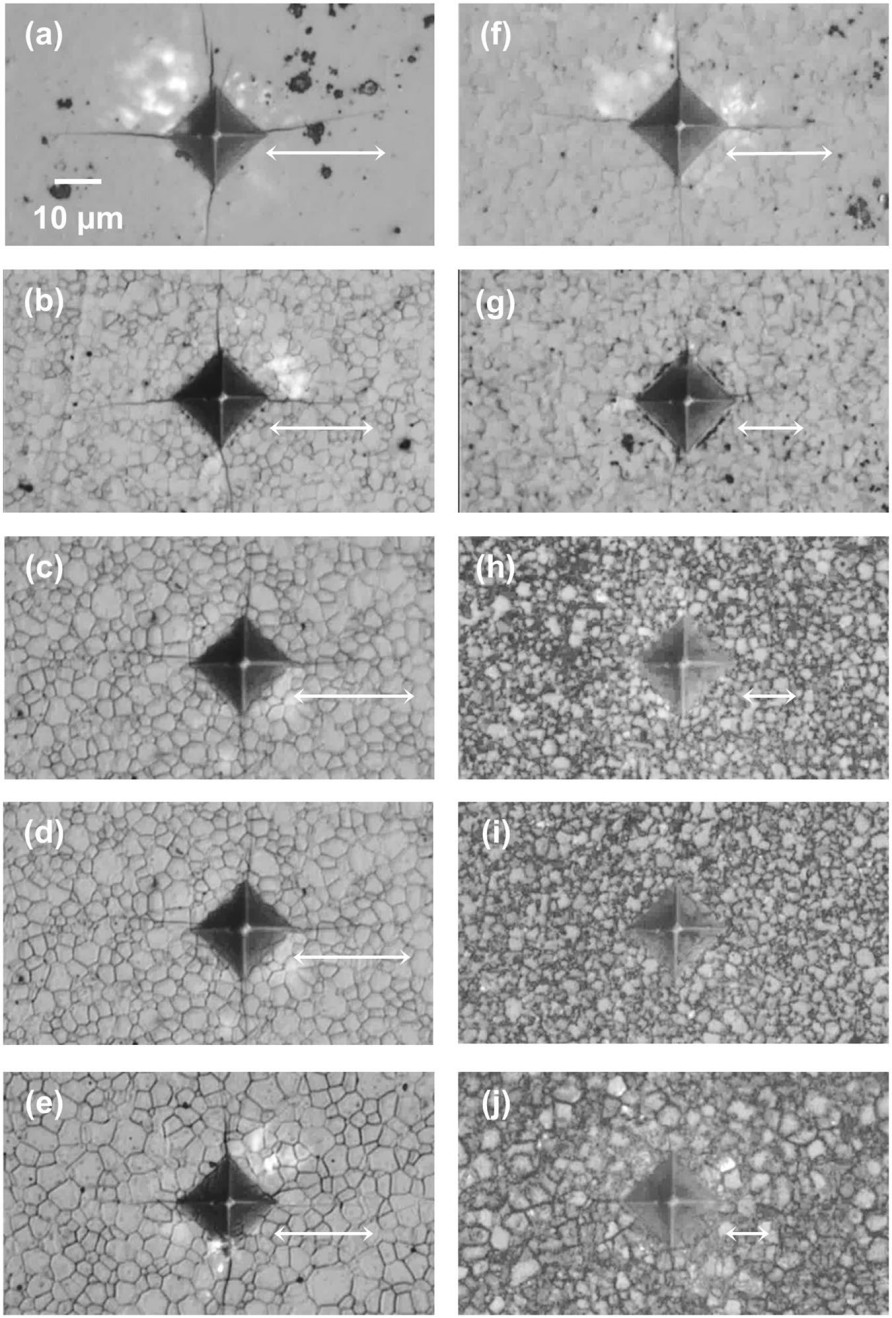
Vickers indents on uncoated (left) and coated (right) areas of 8 YSZ substrate with as-deposited 0.75 μm Y_2_O_3_ coating (**a,f**) and after anneal/quench at 1450 °C for 1 h (**b,g**), 3 h (**c,h**), 6 h (**d,i**), and 12 h (**e,j**), arrows indicate indentation crack lengths (cracks not visible for (**i**), optical micrographs 50x obj.).

Hardness did not change significantly with coating or thermal treatment (Table 1), and was only marginally lower for coated compacts as indents penetrated well beyond coatings (Fig. S3). Cracks emanated linearly from hardness indent corners and were usually unbranched (Fig. 7). Radial cracks were determined to be half-penny shaped by optical microscopy. Overall, indentation cracks were shorter only in coated regions of compacts and considerably shorter only after thermal treatment, being shortest for longer anneals (Fig. 7), in some cases being only visible by scanning electron microscopy (Fig. S4). The apparent fracture toughness (*K*_ICa_) determined from indentation cracks using Eq. 5 increased significantly after thermal treatment, in some instances more than doubling (Table 1).

**Table 1:** Select Mechanical Properties versus Coating Thickness and Thermal Treatment.

Coating Thickness [μm]	Thermal Treatment	Stress [MPa]			*K*_ICa_ [MPa√m]^a^		Vickers Hardness^b^	
		Predicted Coating/Substrate^c^	Y_2_O_3_ Coating^d^	YSZ Substrate^d,e^	Uncoated Area	Coated Area	Uncoated Area	Coated Area
0.5 (10 YSZ)	As-dep. 750 °C	−331/0.07	—	—	1.0 ± 0.06	1.1 ± 0.1	1441 ± 44	1412 ± 22
	1450 °C 0.5 h	−649/0.14	—	—	1.2 ± 0.07	1.4 ± 0.1	1414 ± 47	1413 ± 106
0.75 (8 YSZ)	As-dep. 750 °C	−432/0.14	−96 ± 22	−14 ± 4	1.1 ± 0.06	1.3 ± 0.07	1447 ± 49	1437 ± 19
	1450 °C 1 h	−847/0.26	−762 ± 14	−1 ± 3	1.2 ± 0.06	2.0 ± 0.10	1455 ± 31	1352 ± 21
	1450 °C 3 h	—	−745 ± 15	−18 ± 6	1.1 ± 0.05	2.2 ± 0.2	1510 ± 54	1438 ± 49
	1450 °C 6 h	—	−643 ± 11	11 ± 4	1.3 ± 0.08	2.6 ± 0.8	1482 ± 66	1469 ± 33
	1450 °C 12 h	—	−589 ± 17	12 ± 9	1.2 ± 0.08	3.2 ± 1.1	1483 ± 47	1404 ± 48
1.5 (8 YSZ)	As-dep. 750 °C	−482/0.27	−482 ± 64	−1 ± 2	1.1 ± 0.03	1.8 ± 0.2	1516 ± 46	1382 ± 80 ^f^
	1450 °C 12 h	−847/0.53	−600 ± 7	−2 ± 5	—	—	—	—

^a^Apparent fracture toughness (*K*_ICa_) determined by indentation using the Anstis relation (*E*_YSZ_ 215 GPa).

^b^Vickers hardness with 500 g load; similar trend observed for 200 g and 300 g loads.

^c^Predicted values using Eqs 1–3, which do not account for annealing or stress gradients.

^d^Residual stress from *d* vs sin^2^*ψ* plots using (622) peaks for Y_2_O_3_ and (620) for YSZ (negative indicates compression).

^e^Compressive stress in substrates adjacent to coatings is due to Y^3+^ diffusion and XRD probing near interface.

^f^Lower values for 1.5 μm thick coatings consistent with yttria’s lower hardness.

Dash “—“ indicates values not measured.

**Figure 8:**
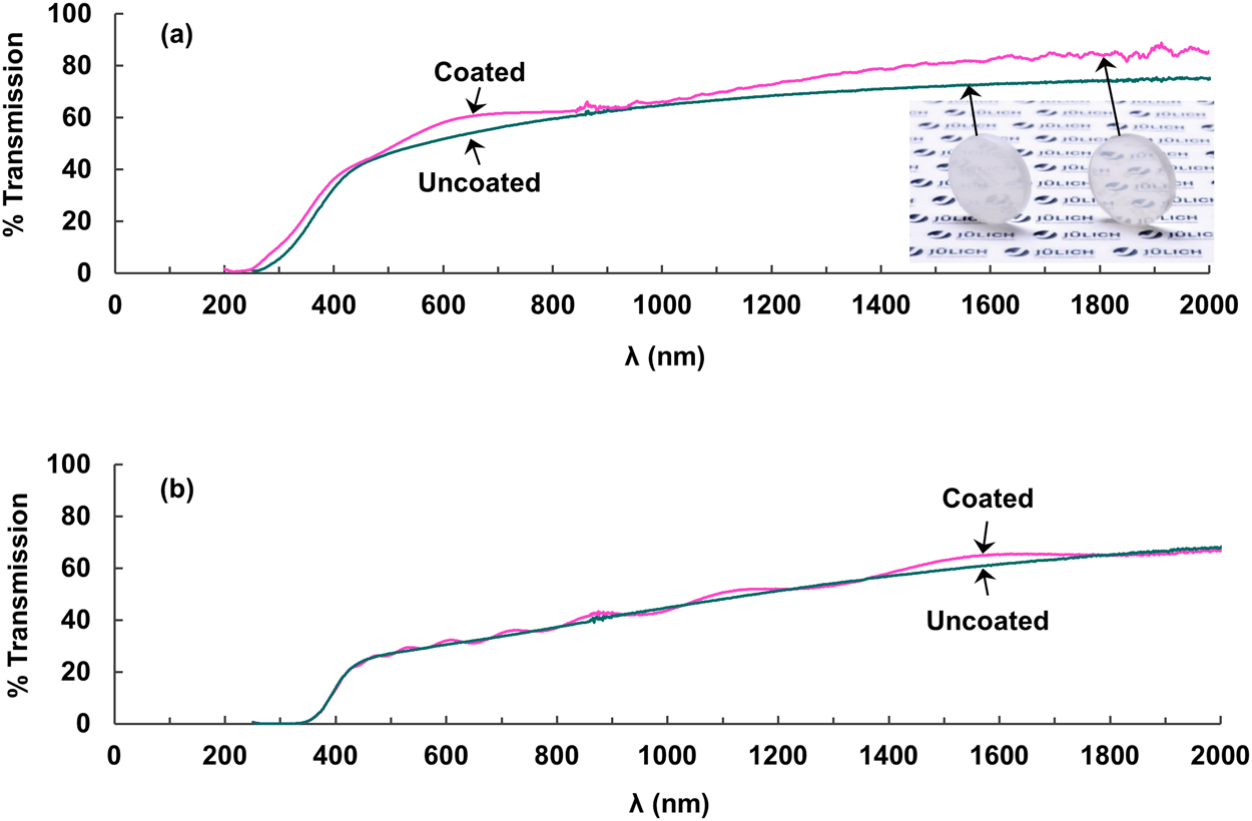
Transmittance for **(a)** 2.3 mm thick 10 YSZ substrate with and without 0.5 μm Y_2_O_3_ coating and **(b)** 2.3 mm thick 8 YSZ substrate with and without 1.5 μm Y_2_O_3_ coating (Y_2_O_3_ coatings applied to both sides of substrates, waviness caused by thickness interference).

For 10 YSZ substrates, total and in-line transmittance were higher, and reflectance lower by several percent after coating (Fig. 8a), as expected from anti-reflection caused by yttria’s lower refractive index^9^. Moreover, thickness interference increased transmittance by up to 5% for some wavelengths. Transmission increase was less significant for coated 8 YSZ substrates, varying only with thickness interference (Fig. 8b). Coating thickness determined from the thickness-interference wavelength corresponded to the thickness determined by scanning and transmission electron microscopy, Calo test, and quartz-crystal microbalance.

## Discussion

Coating compression was confirmed by XRD and suggested by strain contours and dislocations in adjacent substrates, by indentation cracks deviating away from coated areas, and by compressive stresses only being significant after annealing and quenching. Moreover, measured coating and substrate stresses were consistent with calculated values based on their thicknesses and physical properties and the heat treatment temperature differences. Deviations from predicted stresses were attributed to interdiffusion, dislocation-assisted yield near the interface, and stress gradients, which are all unaccounted for by Eqs 1–3. Compressive stress reduction with increasing annealing time combined with increasing apparent fracture toughness was consistent with substrate-coating cation interdiffusion beneficially altering the residual stress field. This trend permitted tailoring additional toughness increase and was clearly demonstrated for the 0.75 µm thick coating, with stresses approaching predictions for shorter anneals and thicker coatings.

Hardness was slightly lower for substrates with thicker (1.5 μm) coatings, consistent with expected lower values for yttria^9,41^. However, indent size did not change appreciably after applying thinner (0.5–0.75 μm) coatings without thermal treatment, indicating the coatings were not shielding substrates from indents. Moreover, minor indentation crack-length differences between coated and uncoated areas of as-coated substrates, as opposed to much shorter cracks in coated versus uncoated areas after thermal treatment indicated genuine surface-compression induced toughness increase. Furthermore, unchanged indentation crack-lengths in uncoated thermally-treated substrates indicated the thermal treatments themselves were not responsible for toughening. Although the Anstis relation does not account for the presence of a compressed layer, surface-compression toughening was conclusively demonstrated, and while toughness values vary with the method used to determine them, the Anstis relation at the least provides a comparative analysis^42^.

Coating-substrate interfaces remained optically-smooth and planar after deposition and thermal treatment, and although grain-growth occurred in coatings, pore growth was not observed. Increased transmittance in coated 10 YSZ substrates was consistent with reduced reflection due to yttria’s lower refractive index. However, transmittance for 8 YSZ substrates was not appreciably increased by the coatings, partly due to lower initial transparency for this stoichiometry. Thickness interference further increased the transmittance of coated substrates for certain wavelengths and this increase was tailorable to specific wavelengths by varying the coating thickness.

The present strategy and achieved toughening enhancement is significant since; (i) coatings and thermal treatment can be applied as a post-treatment, (ii) only a small amount of material is required for coating, (iii) the combined time for coating and thermal-treatment is in the order of hours and is amenable to mass-production, (iv) since the coatings are uniformly thick, post-polishing is not required, (v) compressive stresses generated in the coatings are on the order of those achieved in ion-exchange strengthened glasses, (vi) apparent fracture toughness can in some cases be doubled or even tripled, and (vii) by choosing a coating with a lower refractive index, transmittance can be simultaneously increased.

Although this work is a proof-of-concept, the results strongly suggest applicability in real-world components like damage resistant windows, optical lenses or laser host materials. Surface compression strengthening is widely used in systems ranging from shot-peened steel to tempered glass and ceramics should be no exception. The crux for transparent ceramics is finding a compatible combination of substrate and compressed layer that maximizes mechanical properties while not adversely affecting optical properties during thermal treatment. For the YSZ system, the small hardness decrease (~10–150 HV) associated with using a yttria coating still yields a significant increase over strengthened glass (~1400 HV vs. 650 HV). Moreover, different material combinations, coating thicknesses and/or thermal treatments can be used to maximize either mechanical or optical properties.

## Conclusion

A simple but effective concept for toughening transparent ceramics was proposed and successfully implemented. By depositing thin, transparent coatings with a lower CTE than that of the underlying substrates, residual compressive stresses of up to 750 MPa were generated, and these in turn increased the apparent fracture toughness. Toughness was further improved due to substrate-coating cation counter-diffusion during thermal treatment. Moreover, by selecting coatings with a lower refractive index than substrates, transmittance was simultaneously increased due to reduced reflection. Furthermore, the transmittance increase could be tailored to specific wavelengths by adjusting the coating thickness. Lastly, the technique requires no post-polishing, can be applied as an after-treatment to finished components, and has the potential to improve the properties of other transparent polycrystalline materials, hence widening applications.

## Electronic supplementary material

Supplementary Information

## Data Availability

The datasets generated during and/or analysed during the current study are available from the corresponding author on reasonable request.

## References

[1] Wang SF (2013). Transparent Ceramics: Processing, Materials and Applications. Prog. S. S. Chem..

[2] Goldstein A, Krell A (2016). Transparent Ceramics at 50: Progress Made and Further Prospects. J. Am. Ceram. Soc..

[3] Ikesue A, Aung YL (2008). Ceramic Laser Materials. Nature Photonics..

[4] Penilla EH, Kodera Y, Garay JE (2013). Blue–Green Emission in Terbium-Doped Alumina (Tb:Al_2_O_3_) Transparent Ceramics. Adv. Funct. Mater..

[5] Dolhen M (2018). Nd^3+^ doped transparent tellurite ceramics bulk lasers. Sci. Reports..

[6] Stuer M, Bowen P, Cantoni M, Pecharroman C, Zhao Z (2012). Nanopore Characterization and Optical Modeling of Transparent Polycrystalline Alumina. Adv. Funct. Mater..

[7] Rubat du Merac M, Reimanis IE, Kleebe H-J, Müller MM (2013). Fifty Years of Research and Development Coming to Fruition; Unravelling the Complex Interactions during Processing of Transparent Magnesium Aluminate (MgAl_2_O_4_) Spinel. J. Am. Ceram. Soc..

[8] Patel, P. J., Gilde, G. A., Dehmer P. G., McCauley, J. W. Transparent Armor. The AMPTIAC Newsletter, Advanced Materials and Processes Technology Information Analysis Center, Rome, NY, 4 [3] Fall (2000).

[9] Weber, M. J. Handbook of Optical Materials. 53, 79, 49–187, 242–305 (CRC Press Boca Raton, FL, 2003).

[10] Carter, C. B., Norton, M. G. Ceramic Materials: Science and Engineering. 325 – 332 (Springer, NY, 2007).

[11] Ma X (2018). Pressureless glass crystallization of transparent yttrium aluminium garnet-based nanoceramics. Nature Comm..

[12] Nishiyama N (2017). Transparent polycrystalline cubic silicon nitride. Sci. Reports..

[13] Green DJ (1983). Compressive Surface Strengthening of Brittle Materials by a Residual Stress Distribution. J. Am. Ceram. Soc..

[14] Lakshminarayanan R, Shetty DK (1996). Toughening of Layered Ceramic Composites with Residual Surface Compression. J. Am. Ceram. Soc..

[15] Green DJ, Cai PZ, Messing GL (1999). Residual Stresses in Alumina-Zirconia Laminates. J. Eur. Ceram. Soc..

[16] Rao MP, Sánchez-Herencia AJ, Beltz GE, McMeeking RM, Lange FF (1999). Laminar Ceramics That Exhibit a Threshold Strength. Science.

[17] Cook RF (2005). Toughening of a Cordierite Glass-Ceramic by Compressive Surface Layers. J. Am. Ceram. Soc..

[18] de Portu G, Micele L, Pezzotti G (2006). Laminated Ceramic Structures from Oxide Systems. Comp. B: Engineering.

[19] Bermejo R, Baudín C, Moreno R, Llanes L, Sánchez-Herencia SJ (2007). Processing Optimisation and Fracture Behavior of Layered Ceramic Composites with Highly Compressive Layers. Comp. Sc. and Tech..

[20] Zhang X, Zhou P, Hu P, Han W (2011). Toughening of Laminated ZrB_2_-SiC Ceramics with Residual Surface Compression. J. Eur. Ceram. Soc..

[21] Anselmi-Tamburini U, Woolman JN, Munir ZA (2007). Transparent Nanometric Cubic and Tetragonal Zirconia Obtained by High-Pressure Pulsed Electric Current Sintering. Adv. Funct. Mater..

[22] Tsukuma K, Yamashita I, Kusonose T (2008). Transparent 8 mol% Y_2_O_3_-ZrO_2_ (8Y) Ceramics. J. Am. Ceram. Soc..

[23] Alaniz JE, Perez-Gutierrez FG, Aguilar G, Garay JE (2009). Optical Properties of Transparent Nanocrystalline Yttria-Stabilized Zirconia. Opt. Mat..

[24] Barsoum, M.W. Fundamentals of Ceramics. 356–380 (Taylor and Francis, NY, 2003).

[25] Simeone D (2003). Monoclinic to Tetragonal Semireconstructive Phase Transition of Zirconia. Phys. Rev. B.

[26] Hannink RHJ, Kelly PM, Muddle BC (2000). Transformation Toughening in Zirconia-Containing Ceramics. J. Am. Ceram. Soc..

[27] Krell A, Klimke J, Hutzler T (2009). Transparent Compact Ceramics: Inherent PhysicalIssues. Opt. Mat..

[28] Scott HG (1975). Phase Relationships in the Zirconia-Yttria System. J. Mat. Sci..

[29] Ondik, H. M., McMurdie, H. F. Phase Diagrams for Zirconium and Zirconia Systems. 3, 100–101 (American Ceramic Society, OH, 1998).

[30] H. Hayashi H (2005). Thermal Expansion Coefficient of Ytria Stabilized Zirconia for Various Yttria Contents. S. S. Ionics.

[31] Gy. R (2008). Ion Exchange for Glass Strengthening. Mat. Sci. Eng. B.

[32] Corning, Corning Gorilla Glass 3, NDR Product Information Sheet E_050613, NY, (2013).

[33] Schott Technical Glass Solutions GmbH, Schott Xensation, Cover Product Brochure, Jena, Germany, (2015).

[34] Munro R. G. Elastic Moduli Data for Polycrystalline Ceramics, NIST Interagency/Internal Report 6853, NIST, (Gaithersburg, MD, 2002).

[35] Eigenmann B, Scholtes B, Macherauch E (1989). Grundlagen und Anwendung der Röntgenographischen Spannngsermittlung an Keramiken und Metall-Keramik-Verbundwerkstoffen. Mat.-Wiss. u. Werkstofftech..

[36] Anstis GR, Chantikul P, Lawn BR, Marshall DB (1981). A Critical Evaluation of Indentation Techniques for Measuring Fracture Toughness: I, Direct Crack Measurements. J. Am. Ceram. Soc..

[37] Matsui K, Yoshida H, Y. Ikuhara Y (2008). Grain-boundary Structure and Microstructure Development Mechanism in 2–8 mol% Yttria-Stabilized Zirconia Polycrystals. Acta Mat..

[38] Savoini B, Bellestersos C, Muñoz Santiuste JE, González R, Chen Y (1998). Thermochemical Reduction of Yttria-Stabilized Zirconia Crystals: Optical and Electron Microscopy. Phys. Rev. B.

[39] Xiao R, Zhang S (1998). Yttrium Oxide Films Prepared by Pulsed Laser Deposition. J. Appl. Phys..

[40] Fitzpatrick, M. E. *et al*. Measurement Good Practice Guide No. 52: Determination of Residual Stresses by X-Ray Diffraction. Issue 2 (National PhysicalLaboratory, Teddington, Middlesex, UK, 2005).

[41] Harris, D. C. Materials for Infrared Windows and Domes: Properties and Performance, 17 (SPIE, Bellingham, WA, 1999).

[42] Marshall DB (2015). The Compelling Case for Indentation as a Functional Exploratory and Characterization Tool. J. Am. Ceram. Soc..

[43] Stecura, S. Campbell, W. J. Thermal Expansion and Phase Inversion of Rare-Earth Oxides. Report 5847 (United States Department of the Interior 1961).

