# Supplementary material for: Increasing Fracture Toughness and Transmittance of Transparent Ceramics using Functional Low-Thermal Expansion Coatings
Source: Sci Rep. 2018 Oct 23;8:15644. doi: 10.1038/s41598-018-33919-5 (PMC6199310; doi:10.1038/s41598-018-33919-5)
Supplement: Supplementary file 1 — Supplementary Information [file 41598_2018_33919_MOESM1_ESM.pdf]

Increasing Fracture Toughness and Transmittance of Transparent Ceramics using Functional Low-Thermal Expansion Coatings

Marc Rubat du Merac[1,2], Martin Bram[1], Jürgen Malzbender[1], Mirko Ziegner[1],
Marcin Rasinski[1], Olivier Guillon[1,3]*

Supplementary materials

Figure S1: Pole figures indicating untextured 8 YSZ substrates and preferred {111} orientation in Y_2O_3 coatings.

Figure S2: Example of d versus sin2ψ residual stress analysis plot for 8 YSZ substrate with 0.75 µm Y_2O_3 EB-PVD coating annealed at 1450°C for 3 h and quenched with calculated residual compressive stress of 643 MPa.

Figure S3: Geometry of Vickers indentations and emanating corner cracks.

Figure S4: Vickers indents on **(a)** uncoated and **(b)** coated areas of 8 YSZ substrate with 0.75 µm Y_2O_3 EB-PVD coating after anneal at 1450°C for 1 h followed by quenching (SEM).

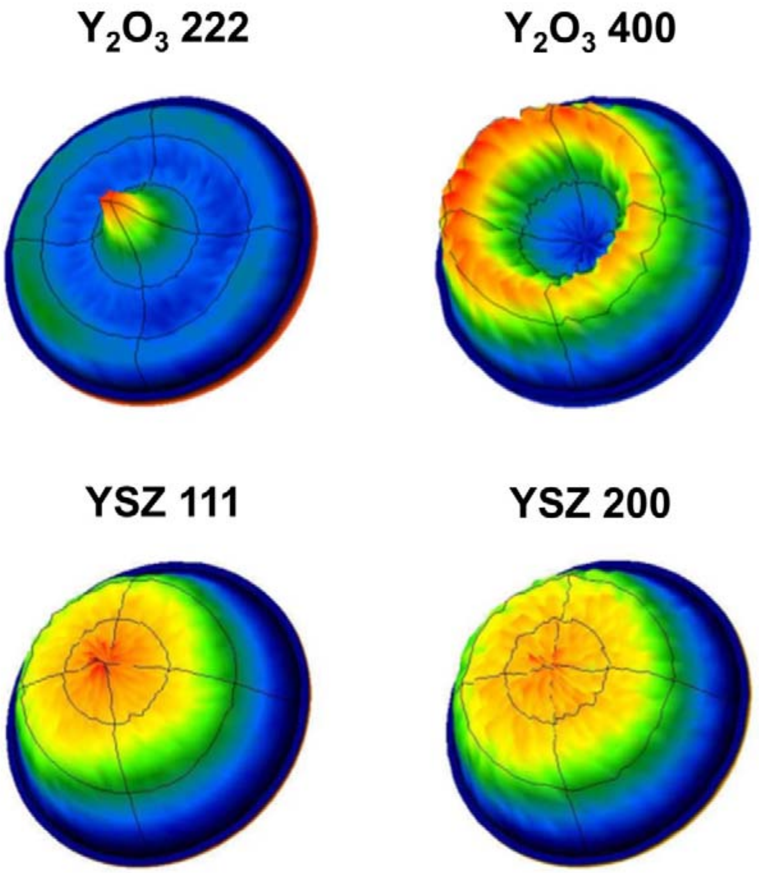

Figure S1: Pole figures indicating untextured 8 YSZ substrates and preferred {111} orientation in Y_2O_3 coatings.

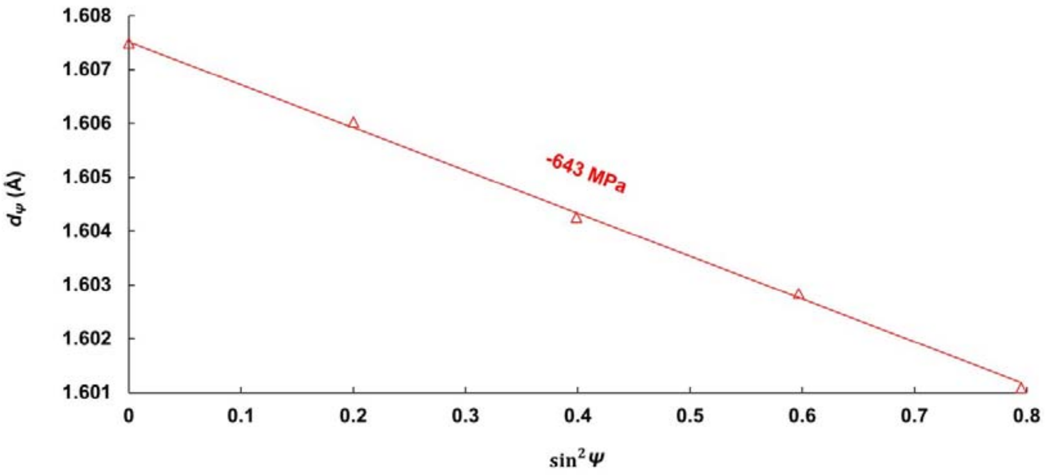

Figure S2: Example of d versus sin2ψ residual stress analysis plot for 8 YSZ substrate with 0.75 μm Y_2O_3 EB-PVD coating annealed at 1450°C for 3 h and quenched with calculated residual compressive stress of 643 MPa.

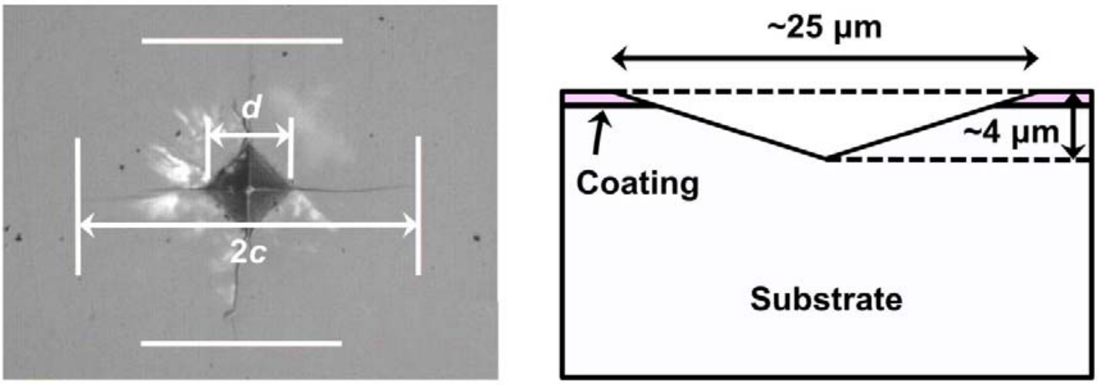

Figure S3: Geometry of Vickers indentations and emanating corner cracks.

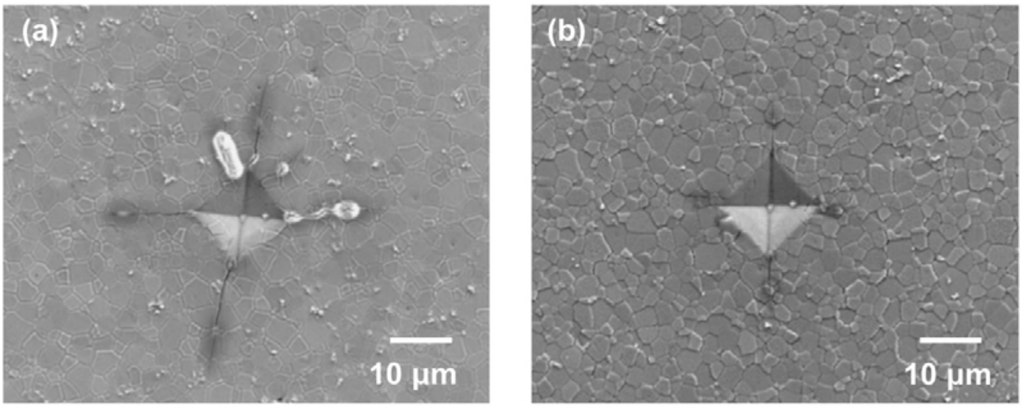

Figure S4: Vickers indents on **(a)** uncoated and **(b)** coated areas of 8 YSZ substrate with 0.75 µm Y_2O_3 EB-PVD coating after anneal at 1450°C for 1 h followed by quenching (SEM).
